# Trauma informed interventions: A systematic review

**DOI:** 10.1371/journal.pone.0252747

**Published:** 2021-06-22

**Authors:** Hae-Ra Han, Hailey N. Miller, Manka Nkimbeng, Chakra Budhathoki, Tanya Mikhael, Emerald Rivers, Ja’Lynn Gray, Kristen Trimble, Sotera Chow, Patty Wilson

**Affiliations:** 1 School of Nursing, The Johns Hopkins University, Baltimore, Maryland, United States of America; 2 Bloomberg School of Public Health, The Johns Hopkins University, Baltimore, Maryland, United States of America; 3 School of Nursing, Duke University, Durham, North Carolina, United States of America; 4 School of Public Health, University of Minnesota, Minneapolis, Minnesota, United States of America; 5 School of Nursing, Vanderbilt University, Nashville, Tennessee, United States of America; 6 Medstar Good Samaritan Hospital, Baltimore, Maryland, United States of America; Koc University School of Medicine, TURKEY

## Abstract

**Background:**

Health inequities remain a public health concern. Chronic adversity such as discrimination or racism as trauma may perpetuate health inequities in marginalized populations. There is a growing body of the literature on trauma informed and culturally competent care as essential elements of promoting health equity, yet no prior review has systematically addressed trauma informed interventions. The purpose of this study was to appraise the types, setting, scope, and delivery of trauma informed interventions and associated outcomes.

**Methods:**

We performed database searches— PubMed, Embase, CINAHL, SCOPUS and PsycINFO—to identify quantitative studies published in English before June 2019. Thirty-two unique studies with one companion article met the eligibility criteria.

**Results:**

More than half of the 32 studies were randomized controlled trials (n = 19). Thirteen studies were conducted in the United States. Child abuse, domestic violence, or sexual assault were the most common types of trauma addressed (n = 16). While the interventions were largely focused on reducing symptoms of post-traumatic stress disorder (PTSD) (n = 23), depression (n = 16), or anxiety (n = 10), trauma informed interventions were mostly delivered in an outpatient setting (n = 20) by medical professionals (n = 21). Two most frequently used interventions were eye movement desensitization and reprocessing (n = 6) and cognitive behavioral therapy (n = 5). Intervention fidelity was addressed in 16 studies. Trauma informed interventions significantly reduced PTSD symptoms in 11 of 23 studies. Fifteen studies found improvements in three main psychological outcomes including PTSD symptoms (11 of 23), depression (9 of 16), and anxiety (5 of 10). Cognitive behavioral therapy consistently improved a wide range of outcomes including depression, anxiety, emotional dysregulation, interpersonal problems, and risky behaviors (n = 5).

**Conclusions:**

There is inconsistent evidence to support trauma informed interventions as an effective approach for psychological outcomes. Future trauma informed intervention should be expanded in scope to address a wide range of trauma types such as racism and discrimination. Additionally, a wider range of trauma outcomes should be studied.

## Background

Despite the United States’ commitment to health equity, health inequities remain a pressing concern among some of the nation’s marginalized populations, such as racial/ethnic or gender minority populations. For example, according to the 2016 National Health and Nutrition Examination Survey (NHANES), 29.1% of Mexican Americans and 24.3% of African Americans with diabetes had hemoglobin A1C greater than 9% (the gold standard of glucose control with levels ≤ 7% deemed adequate), compared to 11% in non-Hispanic whites [1]. The 2016 survey also revealed that 40.9% and 41.5% of Mexican Americans and African Americans with hypertension, respectively, had their blood pressure under control, compared to 51.7% in non-Hispanic whites. In 2014, 83% of all new diagnoses of HIV infection in the United States occurred among gay, bisexual, and other men who have sex with men, with African American men having the highest rates [2].

Several factors have been discussed as root causes of health inequities. For example, Farmer et al. [3] noted structural violence—the disadvantage and suffering that stems from the creation and perpetuation of structures, policies and institutional practices that are innately unjust—as a major determinant of health inequities. According to Farmer et al., because systemic exclusion and disadvantage are built into everyday social patterns and institutional processes, structural violence creates the conditions which sustain the proliferation of health and social inequities. For example, a recent analysis [4] using a sample including 4,515 National Health and Nutrition Examination Survey participants between 35 and 64 years of age revealed that black men and women had fewer years of education, were less likely to have health insurance, and had higher allostatic load (i.e., accumulation of physiological perturbations as a result of repeated or chronic stressors such as daily racial discrimination) compared to white men (2.5 vs 2.1, *p*<.01) and women (2.6 vs 1.9, *p*<.01). In the analysis, allostatic load burden was associated with higher cardiovascular and diabetes-related mortality among blacks, independent of socioeconomic status and health behaviors.

Browne et al. [5] identified essential elements of promoting health equity in marginalized populations such as trauma-informed and culturally competent care. In particular, trauma-informed care is increasingly getting closer attention and has been studied in a variety of contexts such as addiction treatment [6–8] and inpatient psychiatric care [9]. While there is a growing body of the literature on trauma-informed care, no prior review has systematically addressed trauma-informed interventions; one published review of literature [10] limited its scope to trauma survivors in physical healthcare settings. As such, the purpose of this paper is to conduct a systematic review and synthesize evidence on trauma-informed interventions.

For the purpose of this paper, we defined trauma as physical and psychological experiences that are distressing, emotionally painful, and stressful and can result from “an event, series of events, or set of circumstances” such as a natural disaster, physical or sexual abuse, or chronic adversity (e.g., discrimination, racism, oppression, poverty) [11,12]. We aim to: 1) describe the types, setting, scope, and delivery of trauma informed interventions and 2) evaluate the study findings on outcomes in association with trauma informed interventions in order to identify gaps and areas for future research.

## Methods

Five electronic databases—PubMed, Embase, Cumulative Index to Nursing and Allied Health Literature (CINAHL), SCOPUS and PsycINFO—were searched from the inception of the databases to identify relevant quantitative studies published in English. The initial literature search was conducted in January 2018 and updated in June 2019 using the same search strategy.

### Review design

We conducted a systematic review of quantitative evidence to evaluate the effects of trauma informed interventions. Due to heterogeneity relative to study outcomes, designs, and statistical analyses approaches among the included studies, we qualitatively synthesized the study findings. Three trained research assistants extracted study data. Specifically, we used the PICO framework to extract and organize key study information. The PICO framework offers a structure to address the following questions for study evidence [13]: Patient problem or population (i.e., patient characteristics or condition); Intervention (type of intervention tested or implemented); Comparison or control (comparison treatment or control condition, if any), and Outcome (effects resulting from the intervention).

### Eligibility

#### Inclusion criteria

Articles were screened for their relevance to the purpose of the review. Articles were included in this review if the study was: about trauma informed approach (i.e., an approach to address the needs of people who have experienced trauma) or an aspect of this approach, published in English language and involved participants who were 18 years and older. Also, only quantitative studies conducted within a primary care or community setting were included.

#### Exclusion criteria

Exclusion criteria were: studies in or with military populations, refugee or war-related trauma populations, studies with mental health experts and clinicians as research subjects or studies of incarcerated and inpatient populations. Conference abstracts that had limited information on study characteristics were also excluded.

### Search strategy and selection of studies

#### Search strategy

Following consultation with a health science librarian, peer-reviewed articles were searched in PubMed, Embase, CINAHL, SCOPUS and PsycINFO using MeSH and Boolean search techniques. Search terms included: "trauma focused" OR "trauma-focused" OR "trauma informed" OR "trauma-informed." We also searched for the term trauma within three words of informed or focus ((trauma W/3 informed) OR (trauma W/3 focused), or (traumaN3 (focused OR informed)). Detailed search terms for each database are provided in Appendix 1.

#### Study selection

**Fig 1:**
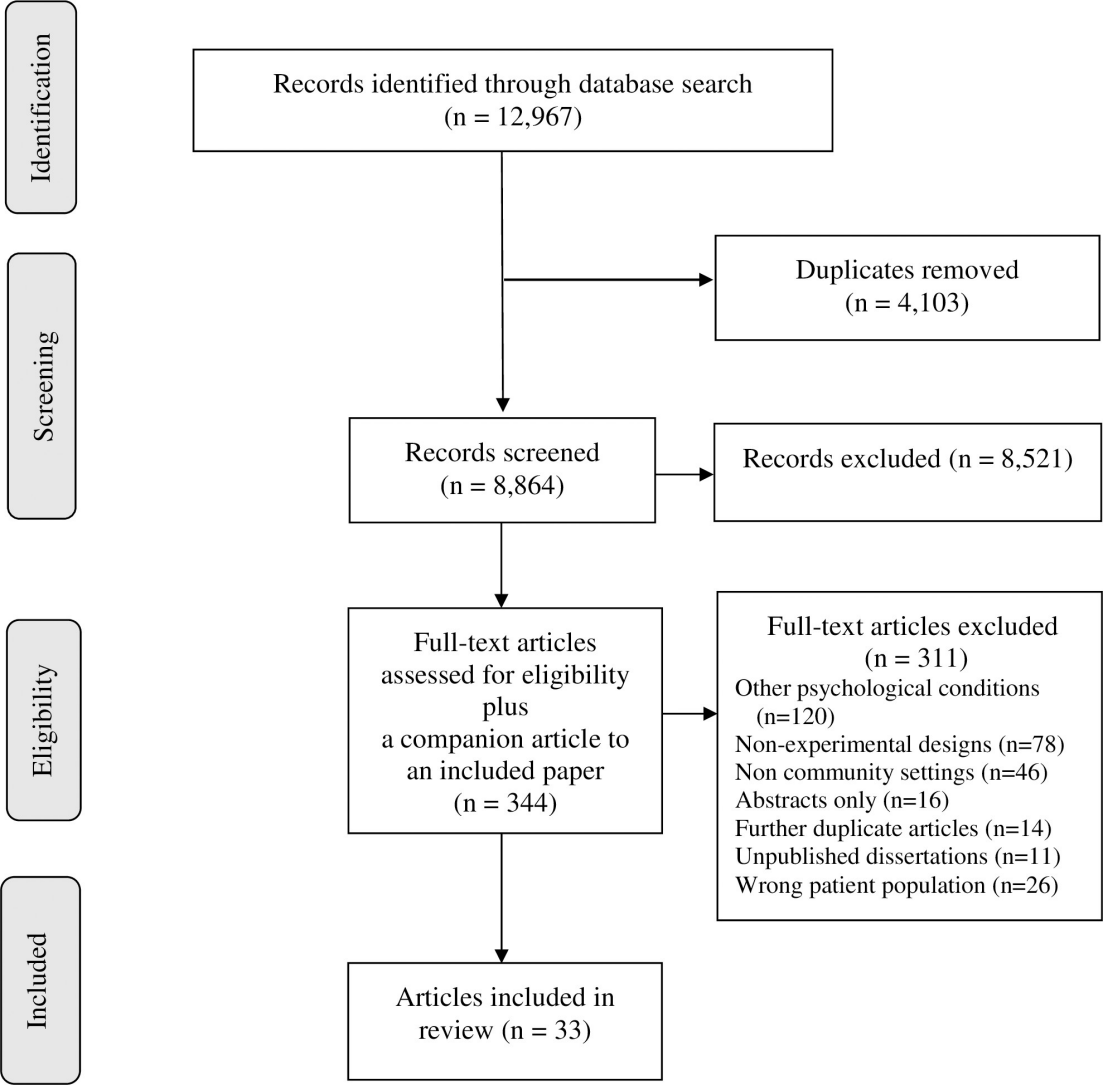
PRISMA diagram of a review of trauma-informed interventions.

The initial electronic search yielded 7,760 references and the follow-up search yielded 5,207 which were all imported into the Covidence software for screening [14]. Screening of the references was conducted by 2 independent reviewers and disagreements were resolved through consensus. There were 4,103 duplicates removed from the imported articles and 8,864 studies were forwarded to the title and abstract screening stage. Eight thousand five hundred and twenty-one studies were excluded because they were irrelevant. Three hundred and forty-three abstracts were identified to be read fully. Following this, 311 articles were excluded for focusing on other psychological conditions (n = 120), were non-experimental studies (n = 78) and were in inpatient or incarcerated populations (n = 46). One additional companion article was identified during full text review. Therefore, thirty-three articles met the inclusion criteria and are reported in this review. Fig 1 provides details of the selection process and identifies the reasons why articles were excluded at each stage.

### Quality assessment

We used the Joanna Briggs Institute quality appraisal tools [15] for randomized controlled trials (RCTs), quasi-experimental studies, and retrospective studies to assess the rigor of each study included in this review. The Joanna Briggs Institute quality appraisal tools [15] include items asking about methodological elements that are critical to the rigor of each type of study designs. In particular, one of the items for RCTs addresses participant blinding to treatment assignment. Due to the nature of trauma-informed interventions included in our review, it was decided that participant blinding is not relevant and hence was removed from the appraisal list for RCTs. No studies were excluded on the basis of the quality assessment. The quality assessment process was conducted independently by two raters. Inter-rater agreement rates ranged from 56% to 100% with the resulting statistic indicating substantial agreement (average inter-rater agreement rate = 77%). Discrepancies between raters were resolved via inter-rater discussion.

## Results

### Overview of studies

Table 1 summarizes the main characteristics of the 32 unique studies included in the review, with one companion article [16] for a study which was later reported with a more thorough examination of findings [17] totaling 33 articles. More than half (n = 19) of the 32 studies were RCTs [17–35] whereas twelve studies were quasi-experimental [36–47] and one was retrospective study [48]. Thirteen studies were conducted in the U.S. [17–19,22,26,27,29,35,39–41,45,47]; five in the Netherlands [30,31,33,38,48]; three in Canada [23,25,46]; two in Australia [21,24]; two in the United Kingdom [36,44]; two in Sweden [42,43]; on study in Chile [20]; Iran [32]; Haiti [37]; South Africa [34]; and Germany [28]. Fourteen of the studies only included females in their sample [18,20,21,23–25,27,28,38–41,45,48]. The average sample size was 78 participants, with a range from 10 participants [38] to 297 participants [48]. Of the studies included, 67% had a sample size above 50 [18–22,26,29–34,36,37,39–42,46–48].

**Table 1:** Characteristics of the studies included in the review.

1^st^ author (yr)[ref]/country	Purpose	Research design/Data points	Sample	Measurement of Trauma	Main outcomes/Measures
Beaumont (2016)[44]/United Kingdom	Investigate the effectiveness of using compassion focused therapy in reducing symptoms of PTSD, anxiety, and depression and increasing self-compassion in fire service personnel	Quasi experimental, a 2×2 mixed-group design with repeated measures/Pre and post intervention	Fire service personnel suffering from PTSD (N = 17; 29% female)	Not directly measured	PTSD symptoms, self-compassion, anxiety and depression/Impact of Events Scale, Self-Compassion Scale–Short Form, Hospital Anxiety and Depression Scale
Booshehri (2018) [18]/United States	Test effectiveness of financial empowerment combined with trauma-informed peer support against standard Temporary Assistance for Needy Families (TANF) programming	RCT/Baseline and follow-up surveys every 3 months over 15 months	Primary caregivers of young children (<6 yrs) and receiving TANF (financial assistance and working at least 20 hours weekly) (N = 103)	Adverse Childhood Experiences and community violence	Family behavioral health, depression, self-efficacy, child’s developmental risks, economic hardship, labor market outcomes**/**Center for Epidemiological Studies—Depression scale, General Self-Efficacy, Parent’s Evaluation of Developmental Status Scale, US Household Food Security Survey Module, Self-reported employment status and hourly earnings
Bowland (2012)[35]/United States	Evaluate the effectiveness of an 11-session, spiritually focused group intervention with older women survivors of interpersonal trauma	RCT/Baseline, at the end of the 11-week intervention, and 3-month follow up	Females age of 55 and older (N = 43)	Self-reported history of ≥ 1 interpersonal traumatic event (child abuse, sexual assault, or domestic violence)	PTSD symptoms, depression, anxiety, somatic symptoms**/**Posttraumatic Stress Diagnostic Scale, Geriatric Depression Scale, Beck Anxiety Inventory, Patient Health Questionnaire
Bryant (2008)[21]/Australia	Determine the efficacy of exposure therapy or trauma-focused cognitive restructuring in preventing chronic PTSD relative to a wait-list control group	RCT/Baseline, immediately post-intervention and 6 months	Trauma survivors (non-sexual or vehicle) meeting diagnostic criteria for Acute Stress Disorder (N = 90)	Not directly measured	Symptoms of acute stress disorder, PTSD, and other psychopathological assessments**/**Acute Stress Disorder Interview, Clinician-Administered PTSD Scale-2, Beck Anxiety Inventory, Beck Depression Inventory, Impact of Event Scale, Posttraumatic Cognition Inventory
Classen (2011)[26]/United States	Compare trauma-focused group psychotherapy with present focused group psychotherapy and a waitlist condition	RCT/Baseline, immediately post-intervention and 6 months	Females with PTSD as a result of childhood sexual abuse (N = 166)	≥ 1 explicit memory of childhood sexual abuse involving genital or anal contact between ages 4-17	PTSD symptoms, total HIV risk, sexual victimization experiences, interpersonal problems**/**PTSD Checklist—Specific, Sexual Experiences Survey, Drug and Alcohol Use Interview, and Sexual Risk Behavior Assessment Schedule, Inventory of Interpersonal Problems-32, Trauma Symptom Inventory, Posttraumatic Growth Inventory
Dalton (2013)[23]/Canada	Examine the impact of emotionally focused therapy on relationship distress in couples in which the female partner had a history of childhood abuse	RCT/Pre and post intervention	Heterosexual couples experiencing clinically significant marital stress (N = 32)	Childhood Trauma Questionnaire, Childhood Maltreatment Interview Schedule	Relationship satisfaction, Therapeutic alliance, Trauma symptoms, childhood trauma symptoms, PTSD symptoms, dissociative experiences**/**Dyadic Adjustment Scale, Couple Therapeutic Alliance Scale, Childhood Trauma Questionnaire, Childhood Maltreatment Interview Schedule, Trauma Symptom Inventory, Dissociative Experience Scale, Couple Therapeutic Alliance Scale
D’Andrea (2012)[45]/United States	Examine the relationship between trauma-focused psychotherapy processes in real-world therapies with complex trauma survivors	Quasi experimental/Pre and post intervention	Females with intimate partner violence (IPV) (N = 27)	Trauma History Questionnaire	PTSD symptoms, dissociative experiences, psychobiological symptoms, general psychiatric distress/Brief Symptom Inventory, Dissociative Experiences Scale, PTSD Checklist, Trauma History Questionnaire, Psychotherapy Process Q set, respiratory sinus arrhythmia, Skin conductance level
Decker (2017)[40]/United States	Describe the impact of a brief, trauma-informed, universal IPV and reproductive coercion assessment and education	Quasi-experimental single group/Baseline, 3 months post-intervention	Females aged 18-36 who had suffered from partner violence (N = 132)	Not directly measured	Interpersonal violence and reproductive coercion/Questions from previous family planning clinic-based studies (Revised Conflict Tactics Scale 2, Perception of Abuse), reproductive coercion measured by 10 questions
Decker (2017)[39]/United States	Develop and test a trauma-informed intervention to improve safety and reduce HIV among female sex workers	Quasi-experimental, single group/Baseline, and 10-12 week follow up	Female sex workers (traded sex for drugs, money, or other resources in the past 3 months; N = 60)	Revised Conflict Tactics Scale adapted for sex work	Depressive symptoms, PTSD symptoms, harm reduction**/**Revised Conflict Tactics Scale, Sex Work-specific Rape Myths Scale, Sex Work Safety Behavior Scale, Condom Confidence scale, Center for Epidemiologic Studies Depression Scale, PTSD CheckList
de Roos (2010)[38]/Netherlands	Test the effectiveness of a trauma focused psychological approach in the treatment of chronic phantom limb pain using a standardized EMDR protocol	Quasi-experimental/2 weeks before and after intervention, 3 mo after intervention and long-term (mean time: 2.8 years)	Individuals with limb amputation from accidents, cancer, medical failures or complex regional pain syndrome (N = 10; 60% female)	EMDR assessment to identify target traumatic memory	Pain intensity, psychological distress, fatigue, PTSD symptoms, health related quality of life/Pain intensity diary, Symptom Checklist 90, Checklist Individual Strength-Revised, Impact of Events Scale and Self-Inventory List, Short Form-36 Health Survey
Doering (2013)[28]/Germany	Investigate the effectiveness of EMDR treatment on reducing dental phobia	RCT/Baseline, 4 weeks, 3 months, and 1 year	Individuals diagnosed with dental phobia (N = 31)	Not directly measured	Dental stress and anxiety/Dental Anxiety Scale, Dental Fear Survey
Dutton (2016)[41]/United States	Examine differential response trajectories to trauma-elated imaginal exposure as a function of affective lability	Quasi-experimental/During sessions	Females with sexual victimization (N = 72)	Sexual victimization that satisfied the definition of a traumatic event as specified in DSM–IV–TR	PTSD symptoms/Clinician-administered PTSD scale, Responses to script-driven imagery scale, Affect Lability Scale-18
Ford (2018)[27]/United States	Test an emotion enhancement to cognitive therapy TARGET (Trauma Affect Regulation: Guide for Education and Therapy)	RCT/Baseline, immediately post-intervention and 1 month follow up	College student problem drinkers and had a history of traumatic childhood stressor or trauma (N = 29)	Traumatic Events Screening Instrument for Adults	Alcohol use and abuse, PTSD, therapy expectancy and working alliance/Global Assessment of Individual Needs-Short Screen alcohol use subscales, Negative Mood Regulation Scale, Stress Reactions Checklist for disorders of extreme stress, PTSD checklist, Expectancy of therapeutic outcome, Brief Working Alliance Inventory
Gawande (2019)[29]/United States	Determine if Mindfulness Training for Primary Care impact health behavior change for primary care patients randomized versus a low-dose comparator	RCT/Baseline, 8-week and 24-week follow up	Individuals with a DSM-V diagnosis (N = 136)	Not directly measured	Behavior change related to self-management of health, anxiety and depressive symptoms, stress, self-emotional regulation, interoceptive awareness, mindfulness, self-compassion/Self-reported level of action plan initiation, Patient-Reported Outcomes Information System (PROMIS) Anxiety and Depression short forms, Perceived Stress Scale, Difficulties in Emotional Regulation Scale, Multidimensional Assessment of Interoceptive Awareness, Five-Facet Mindfulness Questionnaire, Self-Compassion Scale short form, Self-Efficacy for Chronic Disease, Perceived Control Questionnaire
Ginzburg (2009)[22]/United States	Evaluate the effectiveness of group psychotherapy in reducing levels of shame and guilt in adult survivors of childhood sexual abuse at risk for HIV	RCT/Baseline, immediately post-intervention, 6 mo post intervention	Females that experienced childhood sexual abuse between (N = 166; 100% female)	Self-report of ≥ 2 explicit memories of sexual abuse involving genital contact between age 4 -15	Guilt, shame, PTSD**/**Guilt Subscale of the Abuse-Related Beliefs Questionnaire, Shame Subscale of the Abuse-Related Beliefs Questionnaire, Posttraumatic Stress Disorder Checklist
James (2013)[37]/Haiti	Evaluate an evidence-based culturally appropriate lay-person intervention for PTSD experienced by post-Haiti earthquake victims	Quasi-experimental/Pre and post-test	Individuals with PTSD from the 2010 Haitian earthquake (N = 60; 73% female)	Not directly measured	PTSD symptoms, compassion fatigue, posttraumatic growth**/**Harvard Trauma Questionnaire, Professional Quality of Life Scale, Posttraumatic Growth Inventory
Kelly (2015)[16]; (2016)[17]/United States	Evaluate a trauma-informed model of mindfulness-based stress reduction as a phase I trauma intervention for female survivors of IPV	RCT/Baseline and post-intervention	Female IPV survivors (N = 45)	Self-reported history of IPV (physical or sexual abuse)	PTSD symptoms, depressive symptoms, attachment patterns**/**Post-Traumatic Stress Disorder CheckList-Civilian Version, Beck Depression Inventory, Relationship Structures Questionnaire.
Lundqvist (2006)[42]/Sweden	Compare psychological symptoms, symptoms for PTSD, and the sense of coherence across three groups	Quasi-experimental with 3 arms (long-term group therapy, wait list, and short-term group)/Baseline, 12 months (for psychological symptoms and sense of coherence), 2 years after treatment (for inpatient days and sick listing days)	100% Swedish female who were sexually abused in childhood (N = 77; n = 45 for long-term therapy group, n = 10 for wait list group, and n = 22 for short-term therapy group)	Not directly measured	Symptoms of PTSD, psychological symptoms, current psychological health, life attitudes in response to stress, sense of coherence and life events./DSM-IV, Symptom Checklist-90 and Global Severity Index, Sense of Coherence Scale, Life Events
Lundqvist, (2009)[43]/Sweden	Evaluate changes after a two-year-long trauma-focused group therapy program for adult females who had been sexually abused in childhood	Quasi-experimental/Pre and post-test	Female outpatients sexually abused in childhood (N = 45)	Not directly measured	Social interaction, social adjustment, perceived family climate/Interview Schedule of Social Interaction, Social Adjustment scale, Family Climate Test
MacIntosh (2018)[46]/Canada	Describe the implementation of the Skills Training in Affective and Interpersonal Regulation	Quasi-experimental/Pre and post intervention	Individuals that experience childhood sexual abuse (N = 85)	Life Events Checklist for trauma history	Emotion regulation, interpersonal problems, PTSD symptoms/Difficulties in Emotion Regulation Scale, Inventory of Interpersonal Problems, ICD-11 Trauma Questionnaire, Life Events Checklist
Masin-Moyer (2019)[47]/United States	Compare clinical outcomes of a 16-week version of the Trauma Recovery and Empowerment Model (TREM) for women and an attachment-informed adaptation (ATREM)	Quasi-experimental/Pre and post-test	Patients diagnosed with a mental health and/or substance use condition (N = 69; n = 37 in ATREM group, n = 32 in TREM group)	Self-reported history of interpersonal trauma	Group attachment style, perceived social support, difficulty regulating emotions during times of distress, psychological distress related to depression, anxiety, and somatization, PTSD symptoms**/**Relationship Scale Questionnaire, Social Group Attachment Scale, Social Provisions Scale, Difficulties in Emotional Regulation Scale, Brief Symptom Inventory-18, PTSD Symptom Scale, Addiction Severity Index, Life Stressor Checklist–Revised
Matthijssen (2019)[30]/Netherlands	Test if Visual Schema Displacement Therapy is capable of reducing the emotionality and vividness of negative memories	RCT/pre and post intervention	Healthy participants (N = 105; n = 30 in study 1, n = 75 in study 2)	Not directly measured	Emotional disturbances and vividness of traumatic memories**/**Self-report Subjective Units of Disturbance and vividness of the most disturbing part of the memory
Nijdam (2012)[33]/Netherlands	Compare efficacy and response pattern of TF-CBT, brief eclectic psychotherapy for PTSD, with EMDR	RCT/Baseline, weekly at treatment sessions, post-intervention	Individuals 18-65 years with PTSD diagnosis by DSM-IV (N = 70)	Not directly measured	PTSD symptoms, verbal memory, information processing speed, executive functioning**/**Impact of Event Scale–Revised, Structured Interview for PTSD, California Verbal Learning Test, Rivermead Behavioral Memory Test, Trail Making Test, Stroop Color Word Test
Nijdam (2018)[31]/Netherlands	Examine longitudinal changes in neurocognitive functioning before and after trauma-focused psychotherapy	RCT/Assessment before and 17 weeks after start of treatment	Individuals suffering from PTSD (N = 88)	Not directly measured	PTSD symptoms, depressive symptoms, neuropsychological scores**/**Structured Clinical Interview for DSM-IV, Impact of Event Scale–Revised, California Verbal Learning Test, Paragraph Recall Subtest of the Rivermead Behavioral Memory Test, Trail Making Test, Stroop Color Word Test
Nixon (2016)[24]/Australia	Examine the effectiveness of cognitive processing therapy compared with active treatment as usual	RCT/Immediately post-intervention, 3 months, 6 months, and 12 months	Individuals with acute stress disorder that had experienced sexual assault or rape in the past month (N = 47)	Not directly measured	Acute stress disorder and PTSD symptoms**/**Clinician-Administered PTSD Scale, PTSD CheckList, Posttraumatic Cognitions Inventory, MINI International Neuropsychiatric Interview, Beck Depression Inventory-II, Credibility and Expectancy Questionnaire
Noroozi (2018)[32]/Iran	Determine the effectiveness of trauma-based cognitive-behavioral therapy in the treatment of depressed divorced women	Pre/post-test control group/3-month follow up	Females with a history of traumatic event regarding social justice (N = 133)	Not directly measured	Depression symptoms/Beck Depression Inventory
Paivio (2010)[25]/Canada	Evaluate and compare emotion-focused therapy for trauma with imaginal confrontation and emotion-focused therapy for trauma with empathic exploration	RCT/Pre-intervention, mid-intervention, post-intervention and follow up	Individuals that experienced emotional, physical, or sexual childhood abuse (N = 45; 53% female)	Childhood Trauma Questionnaire	PTSD symptoms, interpersonal difficulties, anxiety, depression, self-esteem, resolution and discomfort**/**Impact of Event Scale, State Trait Anxiety Inventory, Beck Depression Inventory-II, Target Complaints (Discomfort) Scale, Rosenberg Self-Esteem Scale, Inventory of Interpersonal Problems, Resolution Scale
Sacks (2008)[19]/United States	Evaluate the effectiveness of the three componentsof the Dual Assessment and Recovery Track program as compared with that of the basic outpatient treatment program	RCT/Baseline, 12 months	Individuals with substance abuse and co-occurring disorders (N = 240)	Trauma History Questionnaire	Substance use, crime, employment, psychological health, trauma, housing, depression, psychological symptoms, community and interpersonal violence, and exposure to trauma**/**Global Appraisal of Individual Needs (GAIN-Q and GAIN-I), Beck Depression Inventory-II, Brief Symptom Inventory, Trauma History Questionnaire
Sikkema (2017)[34] /South Africa	Evaluate feasibility and potential efficacy of the intervention "Improving AIDS Care after Trauma," a coping intervention for HIV-infected women	RCT/Baseline, 3 months, and 6 months	Females with a diagnosis of HIV, met antiretroviral therapy (ART) initiation criteria and had a history of sexual abuse (N = 64)	Self-reported history of sexual abuse	PTSD symptoms, coping (avoidant, spiritual), adherence motivation, HIV care management**/**PTSD CheckList, Life Windows Information-Motivation-Behavioral Skills ART Adherence Questionnaire, HIV Care Engagement survey
Vitriol (2009)[20]/Chile	Examine the effectiveness of a three-month structured outpatient intervention for women with severe depression and childhood trauma	RCT/Baseline, 3 months, 6 months	Females that experience traumatic childhood trauma and have severe depression (N = 87)	Self-reported traumatic experience before age 15; separation from a parent or caregiver, alcohol or drug abuse by family member, physical injury related to punishment, and forced sexual contact	Depressive symptoms, symptoms of PTSD**/**Hamilton Depression Scale, Composite International Diagnostic Interview-10, Lambert’s Outcome Questionnaire-45.2, Post-traumatic Stress Treatment Outcome scale
Nixon (2016)[24]/Australia	Examine the effectiveness of cognitive processing therapy compared with active treatment as usual	RCT/Immediately post-intervention, 3 months, 6 months, and 12 months	Individuals with acute stress disorder that had experienced sexual assault or rape in the past month (N = 47)	Not directly measured	Acute stress disorder and PTSD symptoms**/**Clinician-Administered PTSD Scale, PTSD CheckList, Posttraumatic Cognitions Inventory, MINI International Neuropsychiatric Interview, Beck Depression Inventory-II, Credibility and Expectancy Questionnaire
Noroozi (2018)[32]/Iran	Determine the effectiveness of trauma-based cognitive-behavioral therapy in the treatment of depressed divorced women	Pre/post-test control group/3-month follow up	Females with a history of traumatic event regarding social justice (N = 133)	Not directly measured	Depression symptoms/Beck Depression Inventory
Paivio (2010)[25]/Canada	Evaluate and compare emotion-focused therapy for trauma with imaginal confrontation and emotion-focused therapy for trauma with empathic exploration	RCT/Pre-intervention, mid-intervention, post-intervention and follow up	Individuals that experienced emotional, physical, or sexual childhood abuse (N = 45; 53% female)	Childhood Trauma Questionnaire	PTSD symptoms, interpersonal difficulties, anxiety, depression, self-esteem, resolution and discomfort**/**Impact of Event Scale, State Trait Anxiety Inventory, Beck Depression Inventory-II, Target Complaints (Discomfort) Scale, Rosenberg Self-Esteem Scale, Inventory of Interpersonal Problems, Resolution Scale
Sacks (2008)[19]/United States	Evaluate the effectiveness of the three components of the Dual Assessment and Recovery Track program as compared with that of the basic outpatient treatment program	RCT/Baseline, 12 months	Individuals with substance abuse and co-occurring disorders (N = 240)	Trauma History Questionnaire	Substance use, crime, employment, psychological health, trauma, housing, depression, psychological symptoms, community and interpersonal violence, and exposure to trauma**/**Global Appraisal of Individual Needs (GAIN-Q and GAIN-I), Beck Depression Inventory-II, Brief Symptom Inventory, Trauma History Questionnaire
Sikkema (2017)[34]/South Africa	Evaluate feasibility and potential efficacy of the intervention "Improving AIDS Care after Trauma," a coping intervention for HIV-infected women	RCT/Baseline, 3 months, and 6 months	Females with a diagnosis of HIV, met antiretroviral therapy (ART) initiation criteria and had a history of sexual abuse (N = 64)	Self-reported history of sexual abuse	PTSD symptoms, coping (avoidant, spiritual), adherence motivation, HIV care management**/**PTSD CheckList, Life Windows Information-Motivation-Behavioral Skills ART Adherence Questionnaire, HIV Care Engagement survey
Vitriol (2009)[20]/Chile	Examine the effectiveness of a three-month structured outpatient intervention for women with severe depression and childhood trauma	RCT/Baseline, 3 months, 6 months	Females that experience traumatic childhood trauma and have severe depression (N = 87)	Self-reported traumatic experience before age 15; separation from a parent or caregiver, alcohol or drug abuse by family member, physical injury related to punishment, and forced sexual contact	Depressive symptoms, symptoms of PTSD**/**Hamilton Depression Scale, Composite International Diagnostic Interview-10, Lambert’s Outcome Questionnaire-45.2, Post-traumatic Stress Treatment Outcome scale
Wieferink (2017)[48]/Netherlands	Analyze whether there is a difference in decrease of days of substance use, craving and psychiatric symptoms during subjective units of disturbance treatment between patients with higher or lower levels of PTSD symptoms	Retrospective study/Baseline, 3 months, 6 months	All participants followed regular substance use disorder treatment (N = 297, 72% male)	Not measured	PTSD symptoms, cravings, depression, anxiety and stress**/**Self- Report Inventory for PTSD, Substance Use Inventory, Obsessive Compulsive Drinking Scale, Depression, Anxiety and Stress Scales-21

ART: Antiretroviral Therapy.

ATREM: Attachment-informed adaptation Trauma Recovery and Empowerment Model.

DSM-IV: Diagnostic and Statistical Manual of Mental Disorders 4^th^ edition.

EMDR: Eye Movement Desensitization and Reprocessing.

HIV: Human Immunodeficiency Viruses.

ICD: International Statistical Classification of Diseases and Related Health Problems.

IPV: Intimate Partner Violence.

PTSD: Post-Traumatic Stress Disorder.

RCT: Randomized Controlled Trial.

TANF: Temporary Assistance for Needy Families.

TF-CBT: Trauma-Focused Cognitive Behavioral therapy.

TREM: Trauma Recovery and Empowerment Model.

The studies included in this review recruited their study populations largely based on the type of trauma they were aiming to address, such as individuals that experienced interpersonal traumatic event such as child abuse, sexual assault, or domestic violence [16–18,20–22,24–26,35,40–43,45,46], individuals with substance abuse disorders [19,47,48], couples experiencing clinically significant marital issues [23], individuals with limb amputations [38], dental phobia [28], or fire service personnel suffering from post-traumatic stress disorder [44]. Trauma was self-reported in eight articles [16,17,20,22,26,34,35,47]. In contrast, nine studies clearly identified a measurement of trauma; the Trauma History Questionnaire [19,45], the Childhood Trauma Questionnaire [23,25], the Childhood Maltreatment Interview Schedule [23], the Revised Conflict Tactics Scale adapted for sex work [39], the Traumatic Events Screening Instrument for Adults [27], the Life Events Checklist [46], and the Adverse Childhood Experiences [18]. Two studies used a clinical tool (e.g. eye movement desensitization and reprocessing [38] and Diagnostic and Statistical Manual of Mental Disorders, 4^th^ edition [41] to identify or diagnose trauma. Fifteen studies did not include direct measurements for trauma [21,24,28–33,36,37,40,42–44,48].

### Quality ratings

Tables 2–4 shows final scores of quality assessment. Quality of the 32 unique studies included in this review varied across individual studies. Twelve of 19 RCTs included in the review were of high quality (i.e., 9 to 11) [17,18,20,21,24,26,28,29,31,33–35] and six were of medium quality (i.e., 5 to 8) [19,22,23,25,27,30]. One study scored 4 of 12 [32]. The low rating study [32] lacked relevant information to adequately score its methodological rigor. Most RCTs clearly described randomization, group equivalence at baseline, rates and reasons for attrition, study outcomes, and analysis. Blinding of outcomes assessors to treatment assignment was used and described in several RCTs [17,20,21,24,27,35], whereas blinding of those delivering treatment was discussed clearly in only one study [25]. The majority of the quasi-experimental studies were of high quality (i.e., 7 or higher), except two, which scored 2 of 9 [37] and 6 of 9 [39], respectively. Six of twelve quasi-experimental studies [36,41–44,47] had a comparison group to strengthen internal validity of causal inferences by comparing intervention and control groups. Some of these studies, however, noted differences in baseline assessments between groups [36,43,44]. Finally, one retrospective study [48] scored 11 of 11 and hence was rated as high quality.

**Table 2:** Study quality ratings for randomized control trials.

Randomized controlled trial																			
Items	Boosh-ehri [18]	Bowland [35]	Bryant [21]	Classen [26]	Dalton [23]	Doering [28]	Ford [27]	Gawande [29]	Ginzburg [22]	Kelly [17]	Matth-ijssen [30]	Nijdam [33]	Nijdam [31]	Nixon [24]	Nor-oozi [32]	Paivio [25]	Sacks [19]	Sikk-ema [34]	Vitriol [20]
1. Was true randomization used?	1	1	1	1	?	1	?	1	1	1	?	1	1	1	0	1	1	1	1
2. Was allocation to treatment groups concealed?	1	?	1	1	?	?	?	1	?	?	?	0	0	0	0	?	?	1	?
3. Were treatment groups similar at the baseline?	1	1	1	1	1	1	1	1	0	1	1	1	1	1	0	1	0	1	1
4. Were those delivering treatment blind to treatment assignment?	0	0	0	0	?	?	0	0	?	0	?	0	0	0	0	1	?	0	0
5. Were outcomes assessors blind to treatment assignment?	?	1	1	?	?	?	1	0	?	1	?	0	0	1	0	?	?	?	1
6. Were treatment groups treated identically other than the intervention of interest?	1	1	1	1	1	1	1	1	1	1	1	1	1	1	?	1	1	1	1
7. Was follow up complete and if not, were differences between groups in terms of their follow up adequately described and analyzed?	1	1	1	0	1	1	0	1	1	1	1	1	1	1	?	1	1	1	1
8. Were participants analyzed in the groups to which they were randomized?	1	1	1	1	1	1	1	1	1	1	1	1	1	1	?	1	1	1	1
9. Were outcomes measured in the same way for treatment groups?	1	1	1	1	1	1	1	1	1	1	1	1	1	1	1	1	1	1	1
10. Were outcomes measured in a reliable way?	1	1	1	1	1	1	1	1	1	1	?	1	1	1	1	1	1	1	?
11. Was appropriate statistical analysis used?	1	1	1	1	1	1	1	1	1	1	1	1	1	1	1	?	1	1	1
12. Was the trial design appropriate, and any deviations from the standard RCT design (individual randomization, parallel groups) accounted for in the conduct and analysis of the trial?	1	1	1	1	1	1	1	1	1	1	1	1	1	1	1	?	1	1	1
Total Score	10	10	11	9	8	9	8	10	8	10	7	9	9	10	4	8	8	10	9

**Table 3:** Study quality ratings for quasi-experimental studies.

Quasi-experimental study												
Items	Beaumont[44]	D’Andrea [45]	Decker [40]	Decker [39]	de Jongh [36]	de Roos [38]	Duton [41]	James [37]	Lundqvist [42]	Lundqvist[43]	Mac-Intosh [46]	Masin-Moyer [47]
1. Is it clear in the study what is the ‘cause’ and what is the ‘effect’ (i.e., there is no confusion about which variable comes first)?	1	1	1	1	1	1	1	1	1	1	1	1
2. Were the participants included in any comparisons similar?	0	1	1	0	0	1	1	?	1	0	1	1
3. Were the participants included in any comparisons receiving similar treatment/care, other than the exposure or intervention of interest?	1	1	1	0	1	1	1	0	0	1	1	1
4. Was there a control group?	1	0	0	0	1	0	1	0	1	1	0	1
5. Were there multiple measurements of the outcome both pre and post the intervention/exposure?	1	1	1	1	1	1	1	1	1	1	1	1
6. Was follow up complete and if not, were differences between groups in terms of their follow up adequately described and analyzed?	1	1	1	1	1	1	0	0	1	1	1	1
7. Were the outcomes of participants included in any comparisons measured in the same way?	1	1	1	1	1	1	1	?	1	1	1	1
8. Were outcomes measured in a reliable way?	1	1	?	1	?	1	1	0	1	1	1	1
9. Was appropriate statistical analysis used?	1	0	1	1	1	1	1	0	1	1	1	1
Total Score	8	7	7	6	7	8	8	2	8	8	8	9

**Table 4:** Study quality ratings for cohort study.

Cohort study	
Items	Wieferink [48]
1. Were the two groups similar and recruited from the same population?	1
2. Were the exposures measured similarly to assign people to both exposed and unexposed groups?	1
3. Was the exposure measured in a valid and reliable way?	1
4. Were confounding factors identified?	1
5. Were strategies to deal with confounding factors stated?	1
6. Were the participants free of the outcome at the start of the study (or at the moment of exposure)?	1
7. Were the outcomes measured in a valid and reliable way?	1
8. Was the follow up time reported and sufficient to be long enough for outcomes to occur?	1
9. Was follow up complete, and if not, were the reasons to loss to follow up described and explored?	1
10. Were strategies to address incomplete follow up utilized?	1
11. Was appropriate statistical analysis used?	1
Total Score	11

^+^1 = yes; 0 = no; ? = unclear.

### Characteristics of trauma-informed interventions

#### Type of intervention

Table 5 details the trauma informed intervention characteristics included in this review. The two most frequently used interventions were eye movement desensitization and reprocessing (EMDR) [28,30,31,33,36,38]—a multi-phase intervention using bilateral stimulation, such as left-to-right eyes movements or hand tapping, to desensitize individuals to a traumatic memory or image—and trauma-focused cognitive behavioral therapy or cognitive behavioral therapy (CBT) [26,27,32,46,48]—a psychological approach to introduce emotional regulation and coping strategies (e.g., deep muscle relaxation, yoga, thought discovery and breathing techniques) to deal with negative feelings and behaviors surrounding a trauma of interest [32,48]. The implementation of CBT varied on the trauma of interest. Other studies implemented interventions using general trauma focused therapy [22,43], emotion focused therapy [23,25], stress reduction programs [17], cognitive processing therapy [24], brief electric psychotherapy [31], present focused group therapy [26], compassion focused therapy [44], prolonged exposure [45], stress inoculation training [45], psychodynamic therapy [45], and visual schema displacement therapy [30]. A number of studies included more than one of these therapies [13,26,30,31,33,36,45].

**Table 5:** Trauma-informed intervention characteristics.

1^st^ author (yr)[ref]/Intervention	Intervention Description	Setting	Interventionists	Fidelity	Main Findings
Beaumont (2016)[44]/Compassion focused therapy (CFT) and trauma-focused cognitive behavioral therapy (TF-CBT)	12 weekly sessions of either TF-CBT or TF-CBT with CFT. First and last sessions were 1.5 hours, all others were 1 hour. Both groups received TF-CBT from a psychotherapist and psychoeducation. Those in the TF-CBT group with CFT also received education on the CFT and practiced compassionate letter writing to themselves	Location not specified	Cognitive behavioral therapist	Not addressed	TF-CBT combined with CFT was more effective than TF-CBT alone at increasing self-compassion (p = .05). TF-CBT and CFT not statistically significant for depression and avoidance, however revealed a downward trend in the combined TF-CBT + CFT groups.
Booshehri (2018)[18]/The Building Wealth and Health Network	28-week financial empowerment education with assistance in opening a credit union savings. Matched savings 4-hour weekly peer support group guided by The Sanctuary Model, a trauma-informed approach to social services	Financial empowerment group classes	2 trained financial services organization facilitators	Not addressed	Compared to the other groups, caregivers in the full intervention had better self-efficacy (p = 0.039) and depressive symptoms (p = 0.015) and reduced economic hardship (p = 0.064). Unlike the intervention groups, the control group reported increased developmental risk among their children. Although the control group showed higher levels of employment, the full intervention group reported greater earnings.
Bowland (2012)[35]/Spiritually focused intervention	11 weekly group sessions that manualized psychoeducational cognitive restructuring and skill building approaches to address spiritual struggles in trauma recovery. Sessions were 1.5 hours	Not reported	Not specified	Independent evaluator randomly rated selected videotapes of group sessions	Women in treatment group had lower scores on traumatic, depressive, anxiety and somatic symptoms. Trauma scores fell from mean 19.43 to 11.86. Depressive symptoms decreased by 8.81 compared to .73 increase in control. Anxiety decrease was 6.28 compared to 1.60 increase in control. Somatic symptoms decreased by 2 points compared to increase of 0.55 in control.
Bryant (2008)[21]/Prolonged exposure and cognitive restructuring	5 weekly sessions. Prolonged Exposure consisted of participant engagement in exposure to trauma, homework and strategies to manage stress. Cognitive restructuring consisted of Psychoeducation and homework to restructure thoughts surrounding trauma.	Traumatic Stress Clinic	Master level clinical psychologists	Training manual; weekly supervision of sessions; and audios of 45 random sessions rated by psychologists not involved in intervention	At follow up, patients on prolonged exposure treatment were less likely to meet PTSD criteria than those who underwent cognitive restructuring (37% vs. 63%; odds ratio [OR] = 2.10, 95% confidence interval [CI] = 1.12-3.94) and to achieve full remission (47% vs. 13%; OR = 2.78, 95% CI = 1.14-6.83).
Classen (2011)[26]/Trauma focused group therapy (TFGT) and present focused group therapy (PFGT)	24 weekly sessions (each session lasting 1.5 hours). TFGT involved activation and exploration of trauma memories to restructure cognitive and emotional understanding of traumatic events to minimize the trauma’s impairment on current experience/functioning. PFGT focused on examining current functioning, illuminating in the here-and-now maladaptive expectations and behaviors to help restructure views of self and others.	Research lab	Psychologists, psychiatrists and master level clinicians with prior experience in working with trauma survivors and group therapy	Brief post-session survey completed at end of every session; One randomly selected session for each group rated on the post-session questionnaire by 2 raters who were kept blind to condition	PFGT had greater advantage than TFGT in total HIV risk reduction (p = .05); but all three groups had significant reduction in total HIV risk scores overtime. Both TFGT and PFGT had an advantage on PTSD severity compared to waitlist condition (p*<*.05); but all three groups showed significant reduction in PTSD severity over time. TFGT had a significantly greater reduction in anger/irritability compared with PFGT (p*<*.01).
Dalton (2013)[23]/Emotion focused therapy (EFT)	EFT sessions (22 couple and 2 individual sessions) helped clients symbolize and work through their emotional responses to traumatic events through a focus on creating safe interpersonal connections. Sessions lasted 1.25 hours	Research office	Five therapists, four of which were masters level mental health therapists and one of which was the primary investigator. Therapists had at least 4 years of experience in treating childhood abuse and received five months of weekly training in EFT	All sessions audio-taped. Weekly group supervision done by primary investigator (experienced EFT therapist). A random selection of 25-30% of all taped sessions sent to a senior EFT trainer with 3 therapy implementation check	The couples in EFT group demonstrated significant reduction in relationship distress (p*<*.04). A statistically significant proportion of participants who participated in EFT displayed clinically relevant improvement on the DAS from pretest to post-test (p*<*.001). However, no participants in the control group exhibited clinically significant changes on the DAS.
D’Andrea (2012)[45]/Trauma focused therapy	Trauma focused therapy included prolonged exposure (PE), stress inoculation training (SIT) and psychodynamic therapy (PDT) over 12 weekly sessions. Therapy focused on reconstructing the patient’s memories of the traumatic events by discussing feelings, perceptions, coping, relationships, etc. surrounding the event	Lab setting	22 trauma-oriented therapists who were recruited through printed advertisements or by the clients	Therapists rated their overall treatments using 9 points scales from 1 (extremely uncharacteristic) to 9 (extremely characteristic) via the Psychotherapy Process Q Set	Reduced subjective PTSD symptoms but showed no change in subjective dissociation, depression, anxiety, or interpersonal sensitivity symptoms after 12 weeks. Greater presence of PDT process was significantly associated with greater reductions in PTSD and depression symptoms (p*<*.05 for both). Greater presence of SIT process was related to greater reduction in PTSD symptoms (p*<*.05). Clients showed significant improvement in trauma-related attention bias (p<.01) and anxiety-related attention bias (p*<*.05) but not in attention bias for neutral words. Greater PDT and greater SIT process were both marginally related to reduced implicit memory for anxiety cues (p*<*.1 for both). PE process levels were unrelated to any significant change after 12 weeks of treatment.
Decker (2017)[40]/Trauma informed partner violence assessment with Addressing Reproduction Coercion in Health Settings (ARCHES)	ARCHES assessment included a universal assessment of the recognition of abuse. Intervention included harm reduction counseling and referrals to violence support provider. A provider facilitated discussion of intimate partner violence. Reproductive coercion was addressed with a safety card including suggestions for harm reduction and national resources for violence related help-seeking	Family planning health centers	Physician/Providers who received a day-long training from national experts	Not addressed	Those who received violence related discussion and/or safety resources felt more confident in their providers concern for their safety and ability to respond appropriately to violence. Treatment increased knowledge of violence-related resources. Close to two thirds (65%) of women reported receiving at last one element of the intervention on their exit survey and reported that clinic base Interpersonal violence assessment was helpful irrespective of past violence history.
Decker (2017)[39]/Integrating safety promotion with HIV risk reduction	Brief, semi-structured dialogue that was reinforced with a safety card. Dialogue blended trauma-informed sup- port, validation, safety promotion. Semi-structured dialogue took on average 5–8 minutes and up to 15 minutes depending on participant response and needs. Also linked participants to services.	Mobile vans or adjacent vehicles in community setting	Field research team selected based on experience working with the target population. They underwent training specific to sex workers, violence-related research and practice, and ethics in research	Not addressed	At follow-up, improvements were seen in avoidance of client condom negotiation (p = 0.04) and frequency of sex trade under the influence of drugs or alcohol (p = 0.04). Women’s safety behavior increased (p<0.001). Participants improved knowledge and use of sexual violence support (p<0.01) and use of intimate partner violence support (p<0.01). Change in rape myths, depression and PTSD did not reach statistical significance.
de Jongh (2011)[36]/TF-CBT and Eye Movement Desensitization Reprocessing (EMDR)	TF-CBT: Patient guided through remembrance of trauma via a cohesive narrative of the event(s) until extinction occurs. EMDR: Patients focus on trauma of the event, while tracking a movement with their eyes. *in vivo* exposure involved patient self-managed homework.	Therapist’s office	125 therapists accredited in CBT or EMDR. Patients were assigned to therapist based on geographic proximity	Not addressed	Therapist Rated Outcome revealed that both treatments were highly effective but without significant difference between the treatment groups. Those with travel phobia experienced a greater reduction in symptoms as measured by the Hospital Anxiety Depression Scale (HADS) than those with travel anxiety (p*<*0.04). HADS revealed a significant effect of time but no significance between groups for main effect of treatment or diagnosis.
de Roos (2010)[38]/EMDR	EMDR targeted trauma, pain-related disturbing memories, and phantom-limb pain. Standard EMDR protocol utilized to target trauma and pain-related disturbing memories. Number of sessions individualized to the patient (mean number of sessions 5.9). Sessions lasted 1.5 hours. Sessions occurred weekly.	Individual therapy; location not stated	2 senior psychotherapists trained in EMDR	Not addressed	Significant decrease in pain score (p<0.001) at 2 weeks and 3 months after with an overall time effect for reduction in pain intensity (p<0.02).
Doering (2013)[28]/EMDR	3 weekly sessions. Sessions lasted 1.5 hours. EMDR treatment consisted of reprocessing of memories using the application of eye movements to tax working memory. A series of 25-30 horizontal movements were repeated until the subjective distress reached zero	Psychotherapist’s office at the dental clinical	Therapist trained in both CBT and EMDR. Therapist received specialized EMDR supervision for the treatment of dental phobia	All sessions videotaped. One randomly selected session rated by five different raters	The intervention group improved on all outcome variables except for depression. Dental anxiety total score pretreatment to 12 months (d = 3.28) was significant (p<.001). There was continuing decrease of dental anxiety up to 3 months after treatment and plateaued. Significant reduction of PTSD symptoms between baseline and 3 months follow up (at 12 months, difference was no longer significant).
Dutton (2016)[41]/Imagery exposure	8 trauma focused sessions and 2 neutral session. Sessions were 30 min each and included 5-min baseline exposure and five 5-minute exposure trials. The imagery exposure was conducted with standardized imagery scenes and cued the participants to focus on their active responses (e.g., did your breathing or heart rate change?)	Laboratory	Clinicians (training unspecified)	Not addressed	Mean responses to script-driven imagery scale scores following the first exposure trial were > zero (p<0.001), and symptom ratings decreased significantly across exposure trials (p = 0.001). Past month CAPS score significantly predicted responses to the first trauma script presentation (p<0.001).
Ford (2018)[27]/CBT and Trauma Affect Regulation: Guide for Education and Therapy (TARGET)	8 sessions of manualized internet-supported CBT for problem drinking with or without trauma-focused emotional regulation skills	University of Connecticut counseling center	PhD clinical psychology students received training (10 hours) to conduct both therapies and were randomly assigned to participants. Each therapist conducted at least 5 cases of each therapy modality	First author reviewed therapist’s first two cases and 33% of the sessions following (randomly chosen). Fidelity was achieved on 100% of all items in all sessions in both therapies	Both treatments showed significant reduction in days of alcohol use in the past month (p = .006); days of impairment due to alcohol use were reduced at post-treatment and follow-up only for the CBT+TARGET group but the base rate was very low (approximately 1.25 days in the past month) and the change for both groups was not statistically significant.
Gawande (2019)[29]/Mindfulness Training for Primary Care (MTPC)	MTPC incorporates elements from mindfulness- based stress reduction and mindfulness based cognitive therapy with evidence-based elements from other mindfulness-oriented behavior change approaches. 8 weekly sessions lasting 2 hours and 1 session lasting 7 hours were offered. Participants were recommended to complete 30–45 minutes of daily home practice with guided recordings.	Office for group and home-based practice	13 trained providers including 12 licensed mental health clinicians (e.g., psychology, social-work, psychiatry) and one primary care provider. Providers completed 35 hours of MBSR and 40 hours of MTPC training	Weekly supervision and session-specific fidelity checklists were used. Sessions were audio-recorded and 10% were reviewed by trained observers for adherence and competency, preventing drift	MTPC participants reported a higher rate of action plan initiation (API) compared with low-dose comparator (LDC) of participants who responded to the API survey. MTPC remained associated with higher API. Participants randomized to MTPC, relative to LDC, had significantly higher adjusted odds of self-management action plan initiation in an intention-to-treat analysis (OR = 2.28; 95% CI = 1.02 to 5.06).
Ginzburg (2009)[22]/TFGT	Present focused group therapy (PFGT) focused on the link between symptomatology and the immediate distress. Trauma focused group therapy (TFGT) emphasized the link between symptomology and the past environment. Patients were guided through retrieval and reinterpretation of traumatic memories to work-through and reconstruct painful memories in TFGT and in PFGT, to identify and modify current maladaptive behaviors and coping strategies. Both groups were conducted over 24 weekly sessions (sessions lasted 1.5 hours).	Three universities in California	Licensed clinical psychologists	Not addressed	Both shame and guilt, significant treatment effects were found for TFGT and PFGT compared with waitlist at 12-months (p = 0.01 and p = 0.03, respectively). Shame and guilt were not significantly related to treatment when TFGT compared with PFGT.
James (2013)[37]/Soulaje Lespri Moun (SLM; "Relief for the Spirit" in Haitian Creole)	Drop in program within the internally displaced people camps. 12 group seminars (2 hour-drop in seminars, 3 times a week). Seminars covered earthquake safety, common somatic and emotional responses to stress and trauma, basic relaxation and self-soothing techniques, coping skills, spirituality.	Internally displaced people camps in Port-au-Prince metropolitan area	Earthquake survivors delivered intervention. US and Haitian mental health and psychosocial professionals trained survivors	Not addressed	In the 1st study, lower trauma scores achieved after SLM (p<.01). In the 2^nd^ study, where seminars were offered more frequently and in a more private space, there was a reduction in PTSD symptoms post seminar attendance (p<.001). In 3^rd^ study, there was a reduction of PTSD symptoms post treatment (p<.001).
Kelly (2015)[16]; (2016)[17]/Trauma informed mindfulness-based stress reduction (TI-MBSR)	The 8-week mindfulness course consisted of movement exercises, didactic lecture, and group discussion. Sessions lasted 2 to 2.5 hours. Participants were also asked to practice mindfulness 30-45 minutes a day with provided CD.	Group sessions were in-person, unspecified location. Guided mindfulness was completed at participants location of choice	Licensed clinical social workers	Ensured fidelity using a checklist to document each intervention component as it was delivered during the session (100% adherence)	TI-MBSR group reported significantly greater reductions in posttraumatic stress than the waitlist control group (p = .004, d = .94). TI-MBSR group reported significantly greater decreases in depression than the waitlist control group (p = .006, d = .86). TI-MBSR group reported significantly greater decreases in anxious attachment than the waitlist control group (p = .033, d = .85).
Lundqvist (2006)[42]/Trauma-focused therapy	46 group therapy sessions with a phase-divided structure. Phase 1 was 22 sessions during 5 months, twice a week to help women discuss their childhood sexual abuse narratives and discuss relationships in family of origin. Phase 2 had 15 weekly sessions during 4 months to work through present life. Phase 3 had 9 monthly sessions during 1 year, to work with separation and get used to autonomy. The group therapy model for the short-term group was limited to 20 weekly sessions and including six topics.	Outpatient treatment unit	2 female group leaders did all group sessions in all 10 groups together	Not addressed	No group differences in psychological and PTSD symptoms and sense of coherence. Significant reductions for the study group in the total symptom score and in 8 of 9 scales of Global Severity Index (p<.05); reductions for the short-term group in 4 of 9 subscales (p<.05); and no differences for the wait-list group. A PTSD reduction for the study group, from 87% to 40% (p<.01) but not for the waiting-list group. An increase in sense of coherence for both groups (10-point and 7-point, respectively; p<.05).
Ginzburg (2009)[22]/TFGT	Present focused group therapy (PFGT) focused on the link between symptomatology and the immediate distress. Trauma focused group therapy (TFGT) emphasized the link between symptomology and the past environment. Patients were guided through retrieval and reinterpretation of traumatic memories to work-through and reconstruct painful memories in TFGT and in PFGT, to identify and modify current maladaptive behaviors and coping strategies. Both groups were conducted over 24 weekly sessions (sessions lasted 1.5 hours).	Three universities in California	Licensed clinical psychologists	Not addressed	Both shame and guilt, significant treatment effects were found for TFGT and PFGT compared with waitlist at 12-months (p = 0.01 and p = 0.03, respectively). Shame and guilt were not significantly related to treatment when TFGT compared with PFGT.
James (2013)[37]/Soulaje Lespri Moun (SLM; "Relief for the Spirit" in Haitian Creole)	Drop in program within the internally displaced people camps. 12 group seminars (2 hour-drop in seminars, 3 times a week). Seminars covered earthquake safety, common somatic and emotional responses to stress and trauma, basic relaxation and self-soothing techniques, coping skills, spirituality.	Internally displaced people camps in Port-au-Prince metropolitan area	Earthquake survivors delivered intervention. US and Haitian mental health and psychosocial professionals trained survivors	Not addressed	In the 1st study, lower trauma scores achieved after SLM (p<.01). In the 2^nd^ study, where seminars were offered more frequently and in a more private space, there was a reduction in PTSD symptoms post seminar attendance (p<.001). In 3^rd^ study, there was a reduction of PTSD symptoms post treatment (p<.001).
Kelly (2015)[16]; (2016)[17]/Trauma informed mindfulness-based stress reduction (TI-MBSR)	The 8-week mindfulness course consisted of movement exercises, didactic lecture, and group discussion. Sessions lasted 2 to 2.5 hours. Participants were also asked to practice mindfulness 30-45 minutes a day with provided CD.	Group sessions were in-person, unspecified location. Guided mindfulness was completed at participants location of choice	Licensed clinical social workers	Ensured fidelity using a checklist to document each intervention component as it was delivered during the session (100% adherence)	TI-MBSR group reported significantly greater reductions in posttraumatic stress than the waitlist control group (p = .004, d = .94). TI-MBSR group reported significantly greater decreases in depression than the waitlist control group (p = .006, d = .86). TI-MBSR group reported significantly greater decreases in anxious attachment than the waitlist control group (p = .033, d = .85).
Lundqvist (2006)[42]/Trauma-focused therapy	46 group therapy sessions with a phase-divided structure. Phase 1 was 22 sessions during 5 months, twice a week to help women discuss their childhood sexual abuse narratives and discuss relationships in family of origin. Phase 2 had 15 weekly sessions during 4 months to work through present life. Phase 3 had 9 monthly sessions during 1 year, to work with separation and get used to autonomy. The group therapy model for the short-term group was limited to 20 weekly sessions and including six topics.	Outpatient treatment unit	2 female group leaders did all group sessions in all 10 groups together	Not addressed	No group differences in psychological and PTSD symptoms and sense of coherence. Significant reductions for the study group in the total symptom score and in 8 of 9 scales of Global Severity Index (p<.05); reductions for the short-term group in 4 of 9 subscales (p<.05); and no differences for the wait-list group. A PTSD reduction for the study group, from 87% to 40% (p<.01) but not for the waiting-list group. An increase in sense of coherence for both groups (10-point and 7-point, respectively; p<.05).
Lundqvist, (2009)[43]/Trauma focused group therapy	2-year long trauma focused group therapy. 46 sessions total with phase 1 containing 22 weekly sessions over 5 months, phase 2 containing 15 weekly sessions over 4 months, and phase 3 comprising 9 sessions over 1 year. Sessions were designed to help women tell their childhood sexual-abuse narratives and to discuss relationships within the family. Each participant was the central narrator in 3 sessions during which she could tell the others about sexual details in abuse, feelings of shame, and feelings of guilt.	Outpatient treatment setting	First author was group leader (faculty of Heath and Society at Malmo University) but second group leader was not specified. Both leaders were female	Not addressed	Levels of social interaction significantly improved, with most evident improvements in total score and adequacy of social integration. The effect size values were .55 and .64, respectively. Social adjustment was significantly improved particularly in subscale of work/studies and homework. Effect size values were .53 and .56, respectively. No significant changes in family climate except for the expressed emotion subscale perceived criticism in relation to the partner that showed a reduction.
MacIntosh (2018)[46]/Skills Training in Affective and Interpersonal Regulation (STAIR) treatment	STAIR consisted of 10 weekly group sessions. First five focused on the impact of trauma on emotions and relationships; labeling and identifying feelings; emotion management; and increased capacity to experience positive emotions. The remaining five sessions included identification of trauma-generated interpersonal ‘‘schemas’’ or expectations that impact current relationships; more positive schemas relevant to effective living in the present; skills training in effective assertiveness; increasing flexibility regarding interpersonal expectations; and enhancing compassion for self and others	Clinic	Center therapist trained by the first author over the course of an intensive day-long training session	Clinical director of the center provided weekly supervision and adherence checks over the course of the groups. Standardized materials were developed by the originator of the STAIR model and given to all therapists.	There was significant reduction in Inventory of Interpersonal Problems scores from pre to post treatment, suggesting lower levels of interpersonal problems (p = .002). There was significant reduction in the mean levels of trauma symptoms reported by participants from pre to post treatment (p = .004).
Masin-Moyer (2019)[47]/Trauma Recovery and Empowerment Model (TREM) versus Attachment-informed adaptation TREM (ATREM)	TREM included 16 weekly sessions. Sessions lasted 1.5 hours. ATREM had the same 16 topics as TREM but also had 3 open weeks to add new attachment information involving imagery, arts, fables, group meditation, transitional objects, body tapping and written and verbal feedback. Open weeks were to integrate more processing by pausing content and initiating in-the-moment exploration of relational dynamics and facilitating dyadic and group connections.	Therapy took place at: an outpatient behavioral health facility, a residential substance use treatment, and an outpatient victim services agency	First-author was TREM trained and trained all other clinicians. Each group had at least 1 licensed masters level social worker or counselor. All facilitators participated in training prior to intervention implementation	A facilitator report fidelity checklist was created by 1st author to ensure weekly discussion questions and activities in the TREM manual were addressed	Pre and post intervention results showed statistically significant reductions in individual and group attachment anxiety (p = .03), group attachment avoidance (p<.001), perceived social support (p = .002), emotional regulation capacities (p<.001), psychological distress, depression, anxiety, and PTSD symptom severity (p<.001) for ATREM and TREM. ATREM associated with statistically significant reductions in individual attachment avoidance.
Lundqvist, (2009)[43]/Trauma focused group therapy	2-year long trauma focused group therapy. 46 sessions total with phase 1 containing 22 weekly sessions over 5 months, phase 2 containing 15 weekly sessions over 4 months, and phase 3 comprising 9 sessions over 1 year. Sessions were designed to help women tell their childhood sexual-abuse narratives and to discuss relationships within the family. Each participant was the central narrator in 3 sessions during which she could tell the others about sexual details in abuse, feelings of shame, and feelings of guilt.	Outpatient treatment setting	First author was group leader (faculty of Heath and Society at Malmo University) but second group leader was not specified. Both leaders were female	Not addressed	Levels of social interaction significantly improved, with most evident improvements in total score and adequacy of social integration. The effect size values were .55 and .64, respectively. Social adjustment was significantly improved particularly in subscale of work/studies and homework. Effect size values were .53 and .56, respectively. No significant changes in family climate except for the expressed emotion subscale perceived criticism in relation to the partner that showed a reduction.
MacIntosh (2018)[46]/Skills Training in Affective and Interpersonal Regulation (STAIR) treatment	STAIR consisted of 10 weekly group sessions. First five focused on the impact of trauma on emotions and relationships; labeling and identifying feelings; emotion management; and increased capacity to experience positive emotions. The remaining five sessions included identification of trauma-generated interpersonal ‘‘schemas’’ or expectations that impact current relationships; more positive schemas relevant to effective living in the present; skills training in effective assertiveness; increasing flexibility regarding interpersonal expectations; and enhancing compassion for self and others	Clinic	Center therapist trained by the first author over the course of an intensive day-long training session	Clinical director of the center provided weekly supervision and adherence checks over the course of the groups. Standardized materials were developed by the originator of the STAIR model and given to all therapists.	There was significant reduction in Inventory of Interpersonal Problems scores from pre to post treatment, suggesting lower levels of interpersonal problems (p = .002). There was significant reduction in the mean levels of trauma symptoms reported by participants from pre to post treatment (p = .004).
Masin-Moyer (2019)[47]/Trauma Recovery and Empowerment Model (TREM) versus Attachment-informed adaptation TREM (ATREM)	TREM included 16 weekly sessions. Sessions lasted 1.5 hours. ATREM had the same 16 topics as TREM but also had 3 open weeks to add new attachment information involving imagery, arts, fables, group meditation, transitional objects, body tapping and written and verbal feedback. Open weeks were to integrate more processing by pausing content and initiating in-the-moment exploration of relational dynamics and facilitating dyadic and group connections.	Therapy took place at: an outpatient behavioral health facility, a residential substance use treatment, and an outpatient victim services agency	First-author was TREM trained and trained all other clinicians. Each group had at least 1 licensed masters level social worker or counselor. All facilitators participated in training prior to intervention implementation	A facilitator report fidelity checklist was created by 1st author to ensure weekly discussion questions and activities in the TREM manual were addressed	Pre and post intervention results showed statistically significant reductions in individual and group attachment anxiety (p = .03), group attachment avoidance (p<.001), perceived social support (p = .002), emotional regulation capacities (p<.001), psychological distress, depression, anxiety, and PTSD symptom severity (p<.001) for ATREM and TREM. ATREM associated with statistically significant reductions in individual attachment avoidance.
Lundqvist, (2009)[43]/Trauma focused group therapy	2-year long trauma focused group therapy. 46 sessions total with phase 1 containing 22 weekly sessions over 5 months, phase 2 containing 15 weekly sessions over 4 months, and phase 3 comprising 9 sessions over 1 year. Sessions were designed to help women tell their childhood sexual-abuse narratives and to discuss relationships within the family. Each participant was the central narrator in 3 sessions during which she could tell the others about sexual details in abuse, feelings of shame, and feelings of guilt.	Outpatient treatment setting	First author was group leader (faculty of Heath and Society at Malmo University) but second group leader was not specified. Both leaders were female	Not addressed	Levels of social interaction significantly improved, with most evident improvements in total score and adequacy of social integration. The effect size values were .55 and .64, respectively. Social adjustment was significantly improved particularly in subscale of work/studies and homework. Effect size values were .53 and .56, respectively. No significant changes in family climate except for the expressed emotion subscale perceived criticism in relation to the partner that showed a reduction.
MacIntosh (2018)[46]/Skills Training in Affective and Interpersonal Regulation (STAIR) treatment	STAIR consisted of 10 weekly group sessions. First five focused on the impact of trauma on emotions and relationships; labeling and identifying feelings; emotion management; and increased capacity to experience positive emotions. The remaining five sessions included identification of trauma-generated interpersonal ‘‘schemas’’ or expectations that impact current relationships; more positive schemas relevant to effective living in the present; skills training in effective assertiveness; increasing flexibility regarding interpersonal expectations; and enhancing compassion for self and others	Clinic	Center therapist trained by the first author over the course of an intensive day-long training session	Clinical director of the center provided weekly supervision and adherence checks over the course of the groups. Standardized materials were developed by the originator of the STAIR model and given to all therapists.	There was significant reduction in Inventory of Interpersonal Problems scores from pre to post treatment, suggesting lower levels of interpersonal problems (p = .002). There was significant reduction in the mean levels of trauma symptoms reported by participants from pre to post treatment (p = .004).
Masin-Moyer (2019)[47]/Trauma Recovery and Empowerment Model (TREM) versus Attachment-informed adaptation TREM (ATREM)	TREM included 16 weekly sessions. Sessions lasted 1.5 hours. ATREM had the same 16 topics as TREM but also had 3 open weeks to add new attachment information involving imagery, arts, fables, group meditation, transitional objects, body tapping and written and verbal feedback. Open weeks were to integrate more processing by pausing content and initiating in-the-moment exploration of relational dynamics and facilitating dyadic and group connections.	Therapy took place at: an outpatient behavioral health facility, a residential substance use treatment, and an outpatient victim services agency	First-author was TREM trained and trained all other clinicians. Each group had at least 1 licensed masters level social worker or counselor. All facilitators participated in training prior to intervention implementation	A facilitator report fidelity checklist was created by 1st author to ensure weekly discussion questions and activities in the TREM manual were addressed	Pre and post intervention results showed statistically significant reductions in individual and group attachment anxiety (p = .03), group attachment avoidance (p<.001), perceived social support (p = .002), emotional regulation capacities (p<.001), psychological distress, depression, anxiety, and PTSD symptom severity (p<.001) for ATREM and TREM. ATREM associated with statistically significant reductions in individual attachment avoidance.
Matthijssen (2019)[30]/Visual Schema Displacement Therapy (VSDT) and EMDR	Experiment had a 3 (EMDR, VSDT, and control; 50-minute sessions) by 2 (pre and post intervention) repeated measures design with a follow-up 6-8 days after completion. All participants received all 3 conditions	Anonymized University in The Netherlands	Research assistant (graduate student) trained in VSDT by the originators of VSDT and EMDR by an accredited trainer	Fidelity checks were based upon video recordings that were carried out on a pilot sample to ensure the procedure was carried out properly	In experiment 1, VSDT emotionality scores were higher than EMDR (p<.001) and the control (p<.001), VSDT and EMDR vividness scores were no different (p = 1.00), VSDT vividness scores were higher than the control (p = .02) and EMDR emotionality and vividness scores were higher than the control (p = .02, p = .01). In experiment 2, VSDT emotionality scores were higher than EMDR and the control (p = .001, p<.001). There was no difference in emotionality score between EMDR and the control (p = .08). There were no differences in vividness score between EMDR and the control (p = .83) and between VSDT and EMDR (p = 1.00). The VSDT vividness score was higher than the control (p = .01).
Nijdam (2012)[33]/Brief eclectic psychotherapy and EMDR	Weekly sessions were applied according to the Dutch treatment manual. Sessions lasted 1.5 hrs. EMDR therapy consisted of identification and processing of distressing images of the traumatic events. After patient focused on image with corresponding negative cognition, the patient was asked to follow the therapist’s finger making saccadic movements in alternation with the patient’s own associations. Distress was measured every 5-10 minutes, until distress level was 0 or 1 and then more positive cognition was introduced as it related to target image. Procedure was repeated for other distressing images and treatment sessions were terminated when trauma memory felt neutral.	Outpatient setting	38 psychiatry residents or master’s level clinical psychologists. Therapists received a 3-day level-I training for EMDR and for brief electric psychotherapy.	Therapists received biweekly group supervision. All sessions audiotaped. Treatment adherence protocols developed to rate 6 brief eclectic psychotherapy sessions and three EMDR sessions using an EMDR Fidelity Scale adapted for use with the Dutch EMDR protocol.	Significant, small- to medium-sized improvements in verbal memory, information processing speed, and executive functioning were found after trauma-focused psychotherapy (Cohen’s d = 0.16–0.68). No differences emerged between treatment conditions. Greater PTSD symptom decrease was related to better post-treatment neurocognitive performance (all p<.005). Patients with comorbid depression improved more than patients with PTSD alone on interference tasks (p<.01).
Nijdam (2018)[31]/Trauma-Focused psychotherapy, including brief electric psychotherapy (BEP) and EMDR	EMDR participants received an average of 6.4 weekly sessions lasting 1.5 hours. In EMDR, the most distressing images of the traumatic event are identified and processed. The patient is instructed to focus on the traumatic image and then asked to perform a distractive task of making eye movements until the distress level is 0 or 1. BEP participants received an average of 14.7 weekly sessions lasting 45 minutes. BEP consists of 2 main phases: imaginal exposure and cognitive restricting	Centre for Psychological Trauma at the Academic Medical Centre at the University of Amsterdam	"Independent assessors"	Not addressed	PTSD symptom decrease was significantly correlated with better post-treatment neurocognitive performance (p<.005). Patients with comorbid depression improved more than patients with PTSD alone on interference tasks (p<.01). BEP and EMDR were equally effective at the end of treatment on self-reported PTSD symptoms (mean difference 3.70; 95% Cl = -.6.63 to 14.03; p = .48) and on clinician rated PTSD (mean difference 2.41; 95% CI = -2.10 to 6.92; p = .29.
Nixon (2016)[24]/Cognitive processing therapy (CPT)	CPT included cognitive restructuring techniques and alternative ways of thinking delivered through clinician and worksheets. 6 weekly sessions. Sessions lasted 1.5 hours.	Sexual assault center	Control group implemented by female staff at sexual assault center with a minimum education of a Bachelor of Social Work. No extra study specific training. Intervention therapists received a 3-day workshop and group consultation throughout the trial	Therapy was audiotaped in both conditions. Therapists were rated on important therapeutic factors (i.e., genuineness, warmth, accurate empathy, professional manner) using the same scale	Both CPT and control (treatment as usual) groups demonstrated large reductions in PTSD and depression symptoms following treatment, and these gains were maintained over the course of follow-ups (Cohen’s *d*s for PTSD symptom reductions = 0.76–1.45). Independent assessment of PTSD severity indicated more CPT participants reached good end-state functioning at 12-month follow-up than control participants (50% vs. 31%). Although both treatments were effective, there were some indications that CPT led to better outcomes relative to usual care
Noroozi (2018)[32]/TF-CBT	Sessions (8 weekly) focused on activities, including: feeling identification, deep muscle relaxation, breathing techniques, cognitive adaptive skills, optimism, thought discovery and challenge, cognitive modification, trauma awareness, and emotional response management. Sessions were lasted 1.5 hours.	Group therapy at Mehravar consulting center	Licensed professional	Not addressed	TF-CBT reduced depression symptoms in divorced women compared to control group (p = 0.001) in the post-test and after the three months follow up.
Paivio (2010)[25]/Emotion focused therapy for trauma	A short-term individual modality that targeted disturbances stemming from childhood abuse (total 16-20 sessions). In one version, clients used imaginal confrontation (IC) with the perpetrators of childhood abuse and neglect in an empty chair. In the other version, empathic exploration (EE), clients explored issues with perpetrators exclusively in interaction with the therapist.	Clinic setting	Doctoral and masters level prepared therapists. Therapists received an additional 39 hours of training to implement therapy	Not addressed	A larger proportion of IC compared with EE clients were improved (88% vs. 78%) and recovered (64% vs. 52%) at post-test but the advantage for IC at follow-up was smaller (79% vs. 77% improved; 67% vs. 64% recovered; and 6% vs. 13% deteriorated).
Sacks (2008)[19]/Dual Assessment and Recovery Track (DART)	DART consisted of a modified residential therapeutic community to strengthen identification within the community and had three specific elements: 1) psychoeducational seminar, 2) trauma-informed addictions treatment, and 3) case management	Outpatient substance abuse treatment program	Not specified	Clinical curricula and manuals used to implement intervention	The DART group had better outcomes on measures of psychiatric severity (p = .04) and psychological/emotional problems (p<.001) and on one measure of housing stability (p = .04) compared to the control group (usual care). No group differences on measures of substance use, crime, and employment.
Sikkema (2017)[34]/Improving AIDS Care After Trauma (ImpACT)	ImpACT was designed to reduce traumatic stress and improve HIV care engagement by developing effective strategies for coping with HIV and trauma, and enhancing adherence to antiretroviral therapy (ART) and not intended as a treatment for PTSD. ImpACT included 4 individual sessions and 3 group sessions. Individual sessions lasted roughly 1 hour and group sessions lasted 1.5 hours.	Primary care health clinic	Lay provider (non-specialist in mental health) trained by study PI and coordinator with ongoing supervision by South African clinical psychologist	Quality assurance (QA) worksheets after each session. Work-book activities completed after each session. QA data was reviewed by study coordinator	There was a decrease in ImpACT arm compared to control for avoidance symptoms and hyperarousal (p = 0.01). There was an increase in ART adherence motivation; ImpACT arm reported greater increase in motivation to adhere to ART than control from baseline to 3-month (47.4% vs. 15.4%; p = 0.02). Decrease in avoidant coping and increase in social/spiritual coping and adherence to motivation in both arms.
Vitriol (2009)[20]/Outpatient psychoeducation	Psychiatry sessions that incorporated psychoeducational elements and monitored symptom change, medication adherence, and self-destructive behaviors. Multi-disciplinary team met weekly with therapist to address possible transference or countertransference. Social worker made home visits or telephone calls as needed. Psychiatry sessions occurred monthly.	Outpatient public health clinic	Multidisciplinary team, including psychiatrist and social worker	Not addressed	Significant differences in the intervention group in Hamilton Depression Scale (Ham-D) scores (p<.001) and Outcome Questionnaire (OQ)-45.2 scores (p<.05) at 3 months. Greater proportion of the intervention group had indicators of remission as measured by OQ-45.2 (39% vs. 14%, p<.05) and by Ham-D (22% vs. 5%, p<.05) at 6 months. No group differences in PTSD symptoms.
Wieferink (2017)[48]/CBT and motivational interviewing	CBT therapy centered around registration of thoughts, feelings and behavior concerning participants substance use problem. Medical detoxification provided if needed. Duration and frequency of intervention not reported	Substance use treatment facility for group and individual sessions	Not specified	Not addressed	After 3 and 6 months of substance use disorder (SUD) treatment, there was no group difference in days of substance use. After 6 months of SUD treatment, symptoms of cravings were significantly diminished in both groups (p = 0.003) with those with higher levels of PTSD improving more. For the group with higher levels of PTSD symptoms, depression, anxiety and stress symptoms improved significantly from baseline to 6 months of treatment (p<0.001). Psychiatric symptoms showed a significant improvement between baseline and 6 months of SUD treatment and revealed a significant difference (p<0.001).

API: Action Plan Initiation.

ARCHES: Addressing Reproduction Coercion in Health Settings.

ATREM: Attachment-informed adaptation Trauma Recovery and Empowerment Model.

BEP: Brief Electric Psychotherapy.

CBT: Cognitive Behavioral therapy.

CFT: Compassion Focused Therapy.

CI: Confidence Interval.

CPT: Cognitive processing therapy.

DART: Dual Assessment and Recovery Track.

EE: Empathic Exploration.

EFT: Emotion-Focused Therapy.

EMDR: Eye Movement Desensitization and Reprocessing.

Ham-D: Hamilton Depression Scale.

HIV: Human Immunodeficiency Viruses.

IC: Imaginal Confrontation.

IRT: Antiretroviral Therapy.

LDC: Low-Dose Comparator.

MTPC: Mindfulness Training for Primary Care.

OR: Odds Ratio.

OQ-45.2: Outcome Questionnaire-45.2.

PDT: Psychodynamic Therapy.

PE: Prolonged Exposure.

PFGT: Present Focused Group Therapy.

PTSD: Post-Traumatic Stress Disorder.

QA: Quality Assurance.

SIT: Stress Inoculation Training.

STAIR: Skills Training in Affective and Interpersonal Regulation.

SUB: Substance Use Disorder.

TARGET: Trauma Affect Regulation: Guide for Education and Therapy.

TF-CBT: Trauma-Focused Cognitive Behavioral Therapy.

TFGT: Trauma-Focused Group Therapy.

TI-MBSR: Trauma-Informed Model of Mindfulness-Based Stress Reduction.

TREM: Trauma Recovery and Empowerment Model.

VSDT: Visual Schema Displacement Therapy.

#### Setting, scope, and delivery of intervention

Twenty of the interventions were identified to occur in an outpatient clinic/setting [19–21,24,25,27–29,31–34,36,39,40,42,43,46–48]. Four of the studies took place in a research lab or office [23,26,41,45], one study occurred in the community [17], and one study implemented therapy in three locations, two of which were outpatient and one of which was a residential treatment center [47]. Lastly, one study occurred in internally displaced people’s camps within a metropolitan area in Haiti [37]. The remaining studies did not identify a specific setting [22,35,38,44].

The interventions ranged in length and time, but most often occurred weekly. The longest intervention was done by Lundqvist and colleagues [43], which lasted a total length of 2-years and included 46 sessions. Several other studies included 20 sessions or more [18,22,23,25,26]. The interventions were most commonly delivered by medical professionals, including but not limited to: psychologists or psychiatrists, therapists, social workers, mental health clinicians and physicians [16,17,20–29,33,36,38,39,41,44–47]. The articles frequently noted that the interventionists were masters-level-prepared or higher in their profession [21,23,25–27,33,40,47]. In addition to standard education and licensure, many of the professionals implementing the interventions were required to obtain further training in the therapy of interest [23–25,27–30,33,36,38–40,46,47]. Two studies were identified to be delivered by lay persons [34,37].

Fidelity was addressed in 16 of the included articles [16,19,21,23,24,26–30,33–35,45–47]. The manner in which fidelity was addressed varied by study. Videotaping or audiotaping therapy sessions [21,23,24,28–30,33,35] were most common, followed by deploying regular supervision of the therapy sessions [21,23,27,29,33,46], using a training manual or intervention protocols [19,21,33,46], or having individuals unaffiliated with the study or blind to the intervention rate sessions [21,26,28,35]. Additionally, three articles utilized fidelity checks/checklists to ensure components of the intervention were addressed [16,30,47] or had patients and/or therapists rate therapy sessions [26,34,45]. Finally, one study had quality assurance worksheets completed after each session that were later reviewed by the study coordinator [34].

### Effects of trauma-informed interventions

Trauma-informed interventions were tested to improve several psychological outcomes, such as post-traumatic stress disorder (PTSD), depression, and anxiety. The most frequently assessed psychological outcome was PTSD, which was examined in 23 out of the 32 studies [17,20–27,31,33,35–39,41,42,44–48]. Among the studies that assessed PTSD as an outcome, 11 found significant reductions in PTSD symptoms and severity following the trauma-informed intervention [17,20,21,24,26,28,34,42,45–47], however, one of these studies, which utilized outpatient psychoeducation, did not find significant differences in reduction between the intervention and control group [20]. Trauma-informed interventions that were associated with a significant reduction in PTSD were a mindfulness-based stress reduction program [16], two therapies using the Trauma Recovery and Empowerment Model (TREM) [47], CBT [26,46], EMDR [28], general trauma-focused therapy [42], psychodynamic therapy [45], stress inoculation therapy [45], present-focused therapy [26], and cognitive processing therapy [24]. In addition, an intervention designed to reduce stress and improve HIV care engagement improved PTSD symptoms; however, this intervention was not intended to treat PTSD [34].

Other commonly assessed psychological symptoms, including depression and anxiety, were examined in 16 [17–21,24–26,29,31,32,35,40,44,47,48] and 10 [21,24,25,28,29,35,36,44,47,48] studies, respectively. Among these, trauma-informed interventions were associated with decreased or improved depressive symptoms in 9 studies [17,18,20,21,24,32,35,47,48] and decreased or improved anxiety in 5 studies [21,28,35,47,48]. For example, Vitriol and colleagues found that outpatient psychoeducation resulted in improved depressive symptoms in women with severe depression and childhood trauma [20]. Similarly, Kelly and colleagues found that female survivors of interpersonal violence experienced a significantly greater reduction of depressive symptoms in the intervention group (mindfulness-based stress reduction) compared to the control group [16,17]. Other therapies that resulted in improved depressive symptoms were TREM [47], prolonged exposure therapy [21], CBT [32, 46], psychoeducational cognitive restructuring [35], and financial empowerment education [18]. Cognitive processing therapy similarly resulted in large reductions in depression symptoms, however this reduction was also observed in the control group [24]. The same studies showed that TREM [47], prolonged exposure therapy [21], CBT [48], and psychoeducational cognitive restructuring [35] were associated with improved anxiety. Lastly, in a separate study than the one highlighted above, EMDR was associated with improved anxiety [28].

A select number of the studies found associations between trauma-informed interventions and other psychological outcomes such as attachment anxiety, attachment avoidance, psychiatric symptoms or dental distress. For example, the trauma-informed mindfulness-based reduction program implemented by Kelly and colleagues was associated with a greater decrease in anxious attachment, measured by the Relationship Structures Questionnaire, compared to the waitlist group [17]. Similarly, Masin-Moyer and colleagues found that TREM and an attachment-informed TREM (ATREM) were associated with significant reductions in group attachment anxiety, group attachment avoidance, and psychological distress in women with a history of interpersonal trauma [47]. Additionally, individuals in an outpatient substance abuse treatment program, consisting of psychoeducational seminars and trauma-informed addiction treatment, experienced significantly better outcomes of psychiatric severity, measured by the Global Appraisal of Individual Needs scale, compared to a control treatment group [19]. Doering and colleagues found that EMDR, compared to the control group, was associated with significantly greater improvement in dental stress, anxiety and fear in patients with dental-phobia [28].

There was a series of interpersonal, emotional and behavioral outcomes assessed in the included studies. For example, adult females that were sexually abused in childhood experienced a significant improvement in social interaction and social adjustment after receiving trauma focused group therapy [43]. Similarly, Dalton and colleagues found that couples that received emotion focused therapy experienced a significant reduction in relationship distress [23] and MacIntosh and colleagues found that individuals that received CBT reported lower interpersonal problems post-treatment [46]. Trauma-based interventions were also associated with emotional outcomes. Visual schema displacement therapy and EMDR both were superior to the control treatment in reducing emotional disturbance and vividness of negative memories [30]. In a separate study, CBT was found to reduce levels of emotional dysregulation in individuals that experienced childhood sexual abuse [46]. Lastly, trauma-informed interventions were associated with behavioral outcomes, including HIV risk reduction [26], decreased days of alcohol use [27], and improvements in avoidance of client condom negotiations, frequency of sex trade under influence of drugs or alcohol, and use of intimate partner violence support [40]. Interventions that were associated with these behavioral outcomes included trauma focused and present focused group therapy [26], CBT [27], and a trauma-informed support, validation, and safety-promotion dialogue intervention [40].

### Publication bias

We analyzed three sets of outcome variables for publication bias: PTSD, depression, and anxiety. Based on Begg and Mazumdar test, there was no evidence of publication bias for PTSD (z = 1.55, p = 0.121) and anxiety (z = 0.29, p = 0.769). However, there was some evidence of publication bias for depression (z = 5.19, p<.001). The statistically significant publication bias for depression appears to be mainly due to large effect sizes in Nixon [24] and Bowland [35].

## Discussion

According to our database search, this is the first systematic review to critically appraise trauma-informed interventions using a comprehensive definition of trauma. In particular, our definition encompassed both physical and psychological experiences resulting from various circumstances including chronic adversity. Overall, there was inconsistent evidence to suggest trauma informed interventions in addressing psychological outcomes. We found that trauma-informed interventions were effective in improving PTSD [17,20,21,24,26,28,34,42,45–47] and anxiety [21,28,35,47,48] in less than half of the studies where these outcomes were included. We also found that depression was improved in less than about two thirds of the studies where the outcome was included [17,18,20,21,24,32,35,47,48]. Although limited in the number of published studies included this review, available evidence consistently supported trauma-informed interventions in addressing interpersonal [23,43,46], emotional [30,46], and behavioral outcomes [26,27,40].

Effective trauma informed intervention models used in the studies varied, encompassing CBT, EMDR, or other cognitively oriented approaches such as mindfulness exercises [16,24,26,28,32,35,45,46,48]. In particular, CBT was noted as an effective trauma informed intervention strategy which successfully led to improvements in a wide range of outcomes such as depression [32,48], anxiety [48], emotional dysregulation [46], interpersonal problems [23,46], and risky behaviors (e.g., days of alcohol use) [27]. While the majority of the studies included in the review were focused on interpersonal trauma such as child abuse, sexual assault, or domestic violence [16–18,20–22,24–26,35,40–43,45,46], growing evidence demonstrates perceived discrimination and racism as significant psychological trauma and as underlying factors in inflammatory-based chronic diseases such as cardiovascular disease or diabetes [4]. Future trauma informed interventions should consider a wide-spectrum of trauma types, such as racism and discrimination, by which racial/ethnic minorities are disproportionately affected from [49].

While the majority of the trauma informed interventions were delivered by specialized medical professionals trained in the therapy [16,17,20–29,33,36,38–41,44–47], several of the articles lacked full descriptions of interventionist training and fidelity monitoring [20,22,25,36,38–41,44]. Two studies were identified to be delivered by lay persons [34,37]. There is sufficient evidence to suggest that lay persons, upon training, can successfully cover a wide scope of work and produce the full impact of community-based intervention approaches [50]. Given such, there is a strong need for trauma informed intervention studies to clearly elaborate the contents and processes of lay person training such as competency evaluation and supervision to optimize the use of this approach.

There are methodological issues to be taken into consideration when interpreting the findings in this review. While twenty-three of 32 studies were of high quality [17,18,20,21,24,26,28,29,31,33–36,38,40–48], some studies lacked methodological rigor, which might have led to false negative results (no effects of trauma informed interventions). For example, about one-third (31%) had a sample size less than 50 [17,23–25,27,28,35,38,43,45]. In addition, half of the quasi-experimental studies [37–40,45,46] did not have a comparison group or when they had one, group differences were noted in baseline assessments [36,43,44]. In several studies, therapists took on both traditional treatment and research responsibilities (e.g., delivery of the intervention) [20,25,29,32,33,36,40,46,47], yet blinding of those delivering treatment was discussed clearly in only one study [25]. This dual role is likely to have led to the disclosure of group allocation, hence, threatening the internal validity of the results. Future studies should address these issues by calculating proper sample size a priori, using a comparison group, and concealing group assignments.

### Review limitations

Several limitations of this review should be noted. First, by using narrowly defined search terms, it is possible that we did not extract all relevant articles in the existing literature. However, to avoid this, we conducted a systematic electronic search using a comprehensive list of MeSH terms, as well as similar keywords, with consultation from an experienced health science librarian. Additionally, we hand searched our reference collections, Second, the trauma informed interventions included in this review were implemented to predominantly address trauma related to sexual or physical abuse among women. Thus, our findings may not be applicable to trauma related to other types of incidence such as chronic adversity (e.g., racism or discrimination). Likewise, there were insufficient studies addressing a wider range of trauma impacts such as emotion regulation, dissociation, revictimization, non-suicidal self-injury or suicidal attempts, or post-traumatic growth. Future research is warranted to address these broader impacts of trauma. We included only articles written in English; therefore, we limited the generalizability of the findings concerning studies published in non-English languages. Finally, we used arbitrary cutoff scores to categorize studies as low, medium, and high quality (quality ratings of 0-4, 5-8, and 9+ for RCTs and 0-3, 4-6, 7+ for quasi-experimental studies, respectively). Using this approach, each quality-rating item was equally weighted. However, certain factors (e.g., randomization method) may contribute to the study quality more so than others.

### Conclusions

Our review of 33 articles shows that there is inconsistent evidence to support trauma informed interventions as an effective intervention approach for psychological outcomes (e.g., PTSD, depression, and anxiety). With growing evidence in health disparities, adopting trauma informed approaches is a growing trend. Our findings suggest the need for more rigorous and continued evaluations of the trauma informed intervention approach and for a wide range of trauma types and populations.

## Supporting information

S1 Checklist (DOCX)

S1 Appendix Search strategies. (DOCX)
