# Supplementary material for: Trauma informed interventions: A systematic review
Source: PLoS One. 2021 Jun 22;16(6):e0252747. doi: 10.1371/journal.pone.0252747 (PMC8219147; doi:10.1371/journal.pone.0252747)
Supplement: S1 Appendix — (DOCX) [file pone.0252747.s002.docx]

Appendix 1. Search strategies

1. Pubmed Search

"trauma focused" OR "trauma-focused" OR "trauma informed" OR "trauma-informed"

1. CINAHL

“trauma N3 (focused OR informed)”

1. Embase

“((trauma W/3 informed) OR (trauma W/3 focused))”

1. Psych Info

“trauma N3 (informed OR focused)”

1. SCOPUS

(((trauma W/3 informed) OR (trauma W/3 focused)))

June 2019

1. Pubmed

"trauma focused" OR "trauma-focused" OR "trauma informed" OR "trauma-informed"

Publication dates from 2017/12/31

1. CINAHL

“trauma N3 (focused OR informed)”

Publication dates 2018 and 2019

1. Scopus

ALL (((trauma W/3 informed) OR (trauma W/3 focused))) AND (LIMIT-TO (PUBYEAR, 2019) OR LIMIT-TO (PUBYEAR, 2018))

1. PsychInfo

“trauma N3 (informed OR focused)”

Publication dates 2018 and 2019

1. Embase

(trauma NEAR/3 informed) OR (trauma NEAR/3 focused) AND (2018:py OR 2019:py)
